# *Moringa oleifera* Leaves Protein Enhances Intestinal Permeability by Activating TLR4 Upstream Signaling and Disrupting Tight Junctions

**DOI:** 10.3390/ijms242216425

**Published:** 2023-11-16

**Authors:** Xiaoxue Liu, Chuyu Xi, Wenjie Li, Hairan Su, Hao Yang, Zhongbin Bai, Yang Tian, Shuang Song

**Affiliations:** 1 College of Food Science and Technology, Yunnan Agricultural University, Kunming 650201, China; lxx@stu.ynau.edu.cn (X.L.); xichuyu@stu.ynau.edu.cn (C.X.); liwenjie@stu.ynau.edu.cn (W.L.); suhairan@stu.ynau.edu.cn (H.S.); yanghao@stu.ynau.edu.cn (H.Y.); 2 Yunnan Key Laboratory of Precision Nutrition and Personalized Food Manufacturing, Yunnan Agricultural University, Kunming 650201, China; 2012019@ynau.com.cn; 3 Engineering Research Center of Development and Utilization of Food and Drug Homologous Resources, Ministry of Education, Yunnan Agricultural University, Kunming 650201, China; 4 College of Veterinary Medicine, Yunnan Agricultural University, Kunming 650201, China

**Keywords:** *Moringa oleifera* leaves protein, allergy, intestinal epithelial barrier, intestinal permeability, tight junctions

## Abstract

Changes in intestinal mucosal barrier permeability lead to antigen sensitization and mast cell-mediated allergic reactions, which are considered to play important roles in the occurrence and development of food allergies. It has been suggested that protein causes increased intestinal permeability via mast cell degranulation, and we investigated the effect of camellia *Moringa oleifera* leaves protein on intestinal permeability and explored its role in the development of food allergies. The current study investigated the effect of *M. oleifera* leaves protein on intestinal permeability through assessments of transepithelial electrical resistance (TEER) and transmembrane transport of FITC-dextran by Caco-2 cells. The expression levels of Toll-like receptor 4 (*TLR4)*, *IL-8*, Occludin, Claudin-1, and perimembrane protein family (ZO-1) were detected by real-time PCR and Western blotting. The effect of *M. oleifera* leaves protein on intestinal permeability was verified in mice in vivo. The serum fluorescence intensity was measured using the FITC-dextran tracer method, and the expression of tight junction proteins was detected using Western blotting. The results showed that *M. oleifera* leaves protein widened the gaps between Caco-2 cells, reduced transmembrane resistance, and increased permeability. This protein also reduced the mRNA and protein levels of Occludin, Claudin-1, and ZO-1. Animal experiments showed that intestinal permeability was increased, and that the expression of the tight junction proteins Occludin and Claudin-1 were downregulated in mice. This study shows that *M. oleifera* leaves protein has components that increase intestinal permeability, decrease tight junction protein expression, promote transmembrane transport in Caco-2 cells, and increase intestinal permeability in experimental animals. The finding that *M. oleifera* leaves active protein increases intestinal permeability suggests that this protein may be valuable for the prevention, diagnosis, and treatment of *M. oleifera* leaves allergy.

## 1. Introduction

*Moringa oleifera* (*M. oleifera*), a species in the genus Moringa, in the family Moraceae, is also commonly referred to as oil *M. oleifera* or the drumstick tree [1,2]. Related studies have found that the protein content of *M. oleifera* leaves is 10 times higher than that of milk and that *M. oleifera* leaves contains more gluten, alcoholic protein, albumin, and globulin than milk [3,4,5], making *M. oleifera* leaves a high-quality source of plant protein [6]. However, as *M. oleifera* consumption has increased, it has become apparent that some people experience allergic reactions such as vomiting, diarrhea, and rash after consuming *M. oleifera* leaves, especially fresh leaves [7].

Food intolerances occur through non-immunologic mechanisms (for instance, milk intolerance secondary to lactose deficiency). Allergens are antigenic substances that can stimulate the body to selectively activate immune cells, induce the body to produce specific immunoglobulin E (IgE), and cause allergic reactions, also known as allergens. Most of the plant allergens identified thus far are proteins or glycoprotein substances with a molecular weight of 10 to 100 kDa. Protein allergy is a multifactorial disease that is closely related to mucosal barrier disruption and immune dysfunction, which make an organism more sensitive to an allergen [8,9,10]. The intestinal mucosal barrier is the most important immune defense structure, as it isolates the internal milieu from exogenous substances in the intestine [11,12,13,14]. The most important anatomical basis for the barrier function of the intestinal mucosa is the presence of intact tight junctions between intestinal epithelial cells [15,16,17]. These junctions close the paracellular gaps and allow the selective passage of nutrients such as ions and amino acids [18,19,20,21]. Previous studies [22,23,24] have revealed allergenic plant-derived foods such as peanuts, soybeans, and wheat containing lectins, which may be inextricably linked to allergies [23,25]. Lectins impair the human immune system and increase intestinal permeability and are classified as antinutritional components of foods [26,27,28]. Lectins can bind to intestinal cells, platelets, plasma proteins, muscles, and organs, causing damage to the intestinal mucosa [29], interfering with the digestion and absorption of food [30,31], and causing dysbiosis of the intestinal flora [32,33], thereby damaging the human intestinal and immune systems [34,35,36]. We hypothesized that the proteins (lectins) in *M. oleifera* increase intestinal permeability and that the passage of macromolecules through the intestinal barrier is the main way in which antigen-presenting cells acquire allergens. Thus far, there have primarily been news reports on *M. oleifera* allergy; research on the components, causes, and mechanisms of *M. oleifera* allergy is rare. Most studies have suggested that the main causes of food allergies are proteins in food [37,38,39]. However, no studies have investigated whether *M. oleifera* leaves proteins cause intestinal permeability changes prior to sensitization.

Previous studies by our group have found that *M. oleifera* contains allergens, and that *M. oleifera* leaves proteins can cause allergy in mice with and without adjuvants. However, the mechanism of allergy has remained unclear. Traditional theories suggest that changes in intestinal mucosal barrier permeability lead to antigen sensitization and mast cell-mediated allergic reactions, which are considered to be important in the development of food allergy. This study shows that *M. oleifera* leaves protein has components that increase intestinal permeability, promote transmembrane transport in Caco-2 cells, and increase intestinal permeability in experimental animals. It has been suggested that protein causes increased intestinal permeability via mast cell degranulation, and we investigated whether *M. oleifera* leaves protein regulates intestinal permeability through immune cell-independent epithelial inflammation, leading to *M. oleifera* leaves protein allergies.

## 2. Results

### 2.1. Viability of Caco-2 Cells

**Figure 1:**
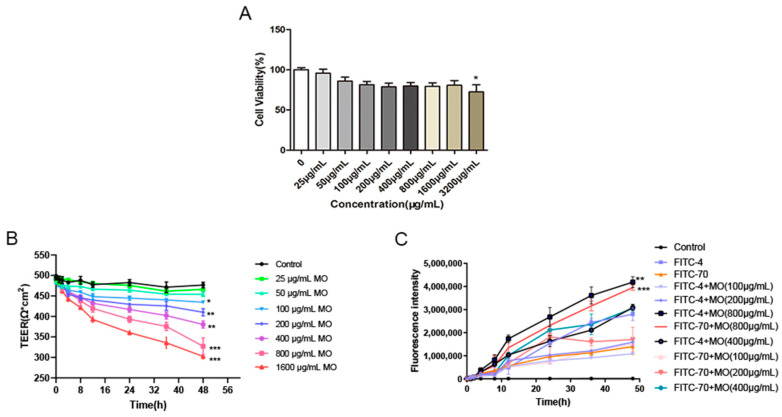
Effects of *M. oleifera* leaves protein on cell viability and cell permeability. (**A**) Effect of *M. oleifera* leaves protein on Caco-2 cell viability as assessed by MTT assay. (**B**) Effect of *M. oleifera* leaves protein on TEER in Caco-2 cells. The data were compared with those of the blank control group (0 μg/mL), * *p* < 0.05, ** *p* < 0.01, *** *p* < 0.001. (**C**) Effect of *M. oleifera* leaves protein on transmembrane transport in Caco-2 cells. Ordinary one-way ANOVA (Dunnett’s multiple comparisons test) was used. Compared with the FITC-4 group, ** *p* < 0.01; compared with the FITC-70 group, *** *p* < 0.001. FITC-4: 1 mg/mL FITC-4; FITC-70: 1 mg/mL FITC-70; FITC-4+MO: 1 mg/mL FITC-4+MO (800 μg/mL); FITC-70+MO: 1 mg/mL FITC-70+MO (800 μg/mL).

Caco-2 cells were treated with 25 μg/mL, 50 μg/mL, 100 μg/mL, 200 μg/mL, 400 μg/mL, 800 μg/mL, and 1600 μg/mL *M. oleifera* leaves protein for 48 h under aseptic conditions. The cell survival rates were 95.82%, 86.07%, 81.39%, 78.76%, 79.81%, 79.40%, 80.78%, and 72.52%, respectively (Figure 1A). Compared with that of the blank control (0 μg/mL) group, the cell viability of the other groups was lower, but there were no significant cytotoxic effects. A significant decrease in cell viability was observed when the concentration reached 3200 μg/mL. Thus, concentrations of 0 μg/mL, 25 μg/mL, 50 μg/mL, 100 μg/mL, 200 μg/mL, 400 μg/mL, 800 μg/mL, and 1600 μg/mL were selected as safe doses for further experiments.

### 2.2. Changes in the TEER of Monolayers of Cells

Caco-2 intestinal epithelial cells were treated in different concentrations of *M. oleifera* leaves protein (25 μg/mL, 50 μg/mL, 100 μg/mL, 200 μg/mL, 400 μg/mL, 800 μg/mL, 1600 μg/mL), and the changes in TEER over time were recorded. After 48 h of treatment, there was basically no change in the TEER value in the blank group. *M. oleifera* leaves protein treatment induced a significant decrease in TEER values in Caco-2 cells. Moreover, the resistance value of caco-2 cells gradually decreased with the increase in *M. oleifera* leaves protein concentration, indicating that M. oleifera leaves protein decreased the monolayer membrane resistance of Caco-2 cells and increased the permeability of Caco-2 cells (Figure 1B).

### 2.3. FITC-Dextran Cellular Transport

FITC transmembrane transport was further examined by detecting the fluorescence intensity of the Transwell fluid. After 48 h treatment of Caco-2 intestinal epithelial cells, there was almost no fluorescence in the blank group. The FITC fluorescence intensity was greater in the FITC-4 group than in the blank group, and the values in the FITC-4+MO and FITC-70+ MO groups gradually increased with time. The fluorescence intensity in the FITC-4+MO group was significantly higher than that in the FITC-4 group (*p* < 0.001); similarly, the value in the FITC-70+MO group was significantly greater than that in the FITC-70 group (*p* < 0.001). These findings further confirmed that *M. oleifera* leaves protein increased FITC transmembrane transport in Caco-2 cells and increased the permeability of Caco-2 cells (Figure 1C).

### 2.4. Effect of M. oleifera Leaves Protein on the mRNA Expression of Tight Junction Proteins in Caco-2 Cells

**Figure 2:**
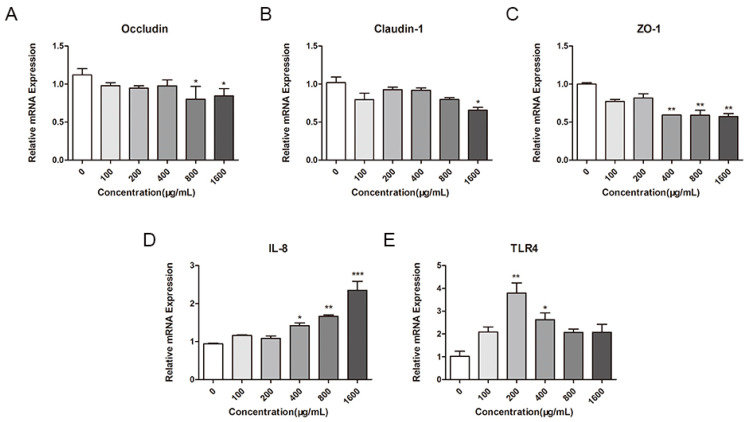
Effects of *M. oleifera* leaves protein on the mRNA expression of tight junction proteins and cytokines in Caco-2 cells. The effects of *M. oleifera* leaves protein on the mRNA expression of the tight junction proteins (**A**) Occludin, (**B**) Claudin-1, and (**C**) ZO-1 were examined in Caco-2 cells. The effects of *M. oleifera* leaves protein on mRNA expression in culture supernatants of Caco-2 cells in response to (**D**) IL-8 and (**E**) TLR4 were examined. The data are presented as the mean ± SD (*n* = 3). Ordinary one-way ANOVA (Dunnett’s multiple comparisons test) was used to compare the treatment groups with the blank control group (0 μg/mL). The significance of differences between groups is represented by *: * *p* < 0.05, ** *p* < 0.01, *** *p* < 0.001.

To further investigate the effect of *M. oleifera* leaves protein on tight junctions in Caco-2 cells, we used fluorescence quantitative RT–PCR. Specifically, we detected the effects of different concentrations of *M. oleifera* leaves protein (100 μg/mL, 200 μg/mL, 400 μg/mL, 800 μg/mL, and 1600 μg/mL) on the mRNA expression of the tight junction proteins Occludin, Claudin-1, and ZO-1 in Caco-2 cells. The results showed that *M. oleifera* leaves protein treatment significantly decreased the mRNA expression of Occludin, Claudin-1, and ZO-1 in Caco-2 cells. As shown in Figure 2, compared with the control treatment (0 μg/mL), treatment with 100 μg/mL, 200 μg/mL, 400 μg/mL, 800 μg/mL, and 1600 μg/mL *M. oleifera* leaves protein reduced the mRNA expression of Occludin in Caco-2 cells by 12.82%, 15.55%, 13.06%, 28.59% (*p* < 0.05), and 24.72% (*p* < 0.05) (Figure 2A); reduced the mRNA expression of Claudin-1 by 21.91%, 9.85%, 10.02%, 21.79%, and 35.73%, respectively (*p* < 0.05) (Figure 2B); and reduced the mRNA expression of ZO-1 by 22.85%, 18.31%, 40.65% (*p* < 0.01), 40.92% (*p* < 0.01), and 42.74% (*p* < 0.01), respectively (Figure 2C). IL-8 is a typical inflammatory cytokine involved in mediating and promoting inflammatory responses [40]. *M. oleifera* leaves protein induced the overexpression of inflammatory cytokines in Caco-2 cells and significantly and concentration-dependently increased the mRNA expression of IL-8 in Caco-2 cells compared with the control group (0 μg/mL); additionally, *M. oleifera* leaves protein treatment can significantly increase the mRNA expression of TLR4 in Caco-2 cells, as TLR4 may happen to be the target gene that activates the signaling pathway (Figure 2D,E).

### 2.5. Effects of M. oleifera Leaves Protein on the Expression of Tight Junction Proteins in Caco-2 Cells

**Figure 3:**
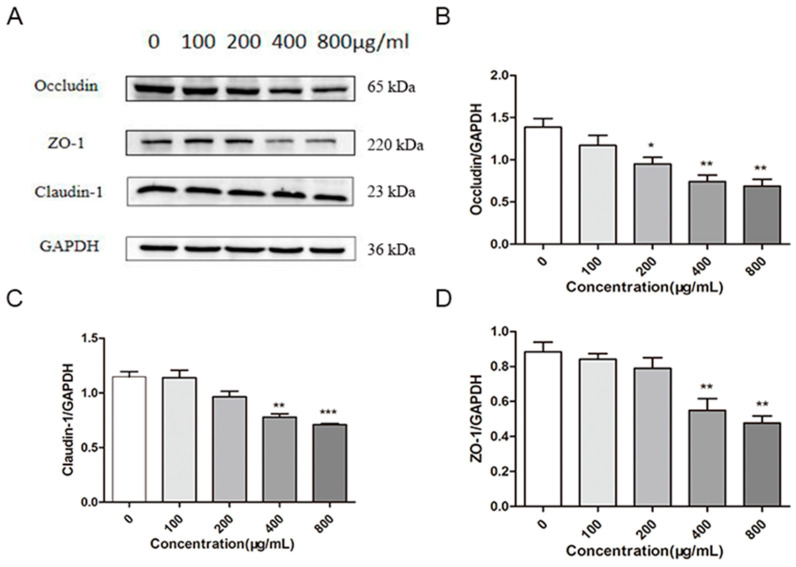
Effects of *M. oleifera* leaves protein on the expression of tight junction proteins in Caco-2 cells. (**A**) Representative Western blot and quantification of the tight junction proteins Occludin (**B**), Claudin-1 (**C**), and ZO-1 (**D**). The data are presented as the mean ± SD (*n* = 3). The data were compared with those of the blank control group (0 μg/mL), and the significance of differences between groups is represented by *: * *p* < 0.05, ** *p* < 0.01, *** *p* < 0.001.

The effects of different concentrations (100 μg/mL, 200 μg/mL, 400 μg/mL, and 800 μg/mL) of *M. oleifera* leaves protein on the expression of the tight junction proteins Occludin, Claudin-1, and ZO-1 in Caco-2 cells were further investigated by protein immunoblotting (Figure 3). The expression of these proteins was reduced by *M. oleifera* leaves protein compared with the blank control (0 μg/mL) in a dose-dependent manner, with concentrations of 100 μg/mL, 200 μg/mL, 400 μg/mL, and 800 μg/mL reducing the expression of Occludin by 12%, 32% (*p* < 0.05), 49% (*p* < 0.01), and 53% (*p* < 0.01), respectively (Figure 3B); reducing the expression of Claudin-1 by 6%, 16%, 32% (*p* < 0.01), and 38% (*p* < 0.001), respectively (Figure 3C); and reducing the expression of ZO-1 by 3%, 16%, 53% (*p* < 0.01), and 54% (*p* < 0.01), respectively (Figure 3D). The results indicated that *M. oleifera* leaves protein reduced the expression of tight junction proteins in Caco-2 cells, which led to an increase in cell permeability.

### 2.6. Effect of M. oleifera Leaves Protein Treatment on Occludin Protein Localization in Caco-2 Cells

**Figure 4:**
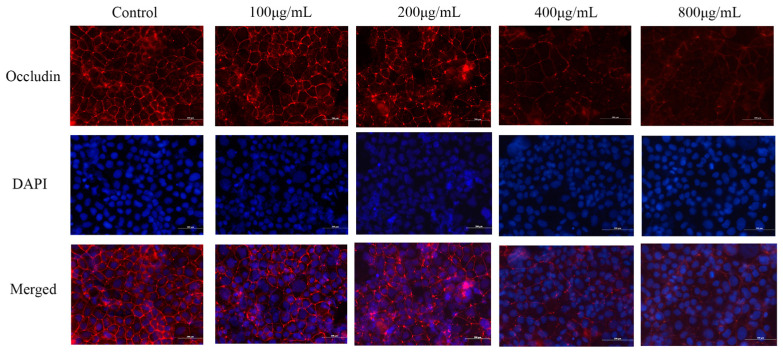
Effect of *M. oleifera* leaves protein treatment on occludin protein localization in Caco-2 cells. Scale bars = 200 μm. As the concentration of *M. oleifera* leaves protein increased, Occludin protein decreased in Caco-2 cells.Treatment with 100 μg/mL, 200 μg/mL, 400 μg/mL, and 800 μg/mL *M. oleifera* leaves protein resulted in a weakened fluorescence intensity and obvious breakage at the junctions.

The protein distribution of Occludin was detected by immunofluorescence, and the results were collected by fluorescence microscopy (Figure 4). The Occludin staining in tight junctions was smoother and more continuous in the control group than in the other groups, and the fluorescence signal was distributed at the cell membrane junctions. Treatment with 100 μg/mL, 200 μg/mL, 400 μg/mL, and 800 μg/mL *M. oleifera* leaves protein resulted in a weakened fluorescence intensity and obvious breakage at the junctions. The most significant effect was observed in the group treated with the highest concentration of *M. oleifera* leaves protein (800 μg/mL). The results indicated that *M. oleifera* leaves protein could reduce the distribution of the tight junction protein Occludin in Caco-2 cells, which led to an increase in cell permeability.

### 2.7. Effects of M. oleifera Leaves Protein on Organ Indices in Mice

**Figure 5:**
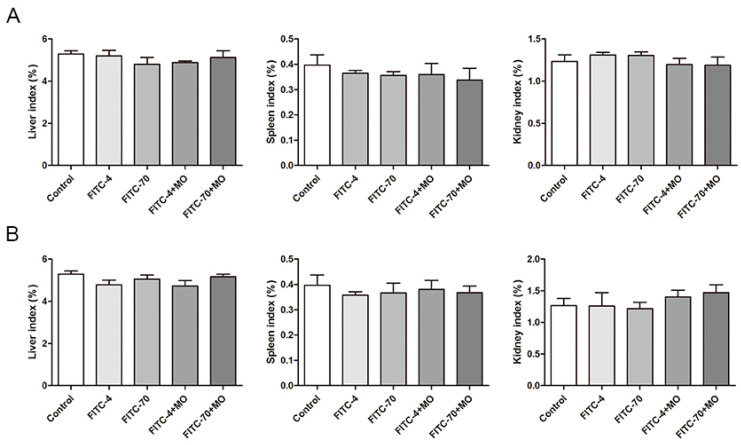
Effects of *M. oleifera* leaves protein on organ indices in mice after (**A**) Effects of *M. oleifera* leaves protein treatment for 4 h on organ indices in mice. (**B**) Effects of *M. oleifera* leaves protein treatment for 8 h on organ indices in mice. The data are presented as the mean ± SD (*n* = 10).

The mice were active and responsive throughout the experiment after gavage, with shiny hair and dark brown stools. After executing the mice, the intestinal tissues were generally normal, without ulceration or inflammation. As shown, the liver indices of the mice in each experimental group were slightly but non-significantly reduced after 4 h of gavage, whereas the spleen and kidney indices were almost unchanged, indicating that FITC-4, FITC-70, and *M. oleifera* leaves protein had no toxic effects on the mice (Figure 5A). As shown after gavage with drugs for 8 h, the kidney indices of the mice in each experimental group were slightly but non-significantly increased, and the liver and spleen indices were almost unchanged. These findings indicated that FITC-4, FITC-70, and *M. oleifera* leaves protein had no toxic effects on the mice after treatment for 8 h (Figure 5B).

### 2.8. Serum Fluorescence Intensity in Mice

**Figure 6:**
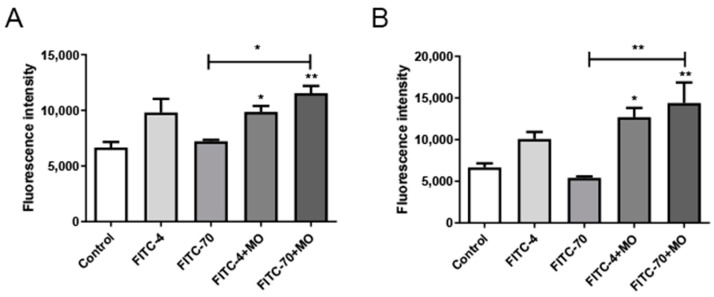
Effects of treatment with *M. oleifera* leaves protein on serum fluorescence intensity. (**A**) Changes in serum fluorescence intensity after 4 h of treatment with *M. oleifera* leaves protein. Compared with the blank group, the FITC-4+MO group had a significant difference (* *p* < 0.05), the FITC-70+MO group had a significant difference (** *p* < 0.01). Compared with the FITC-70 group, fluorescence intensity was significantly increased in FITC-70+MO group (* *p* < 0.05). (**B**) Changes in serum fluorescence intensity after 8 h of treatment with *M. oleifera* leaves protein. Compared with the blank group, the FITC-4+MO group had a significant difference (* *p* < 0.05), the FITC-70+MO group had a significant difference (* *p* < 0.05 or ** *p* < 0.01). Compared with the FITC-70 group, fluorescence intensity was significantly increased in FITC-70+MO group (** *p* < 0.01). The data are presented as the mean ± SD (*n* = 10). Ordinary one-way ANOVA (Dunnett’s multiple comparisons test) was used. FITC-4: 10 mg/mL FITC-4; FITC-70: 10 mg/mL FITC-70; FITC-4+MO: 10 mg/mL FITC-4+MO (80 mg/mL); FITC-70+MO: 10 mg/mL FITC-70+MO (80 mg/mL).

The effect of *M. oleifera* leaves protein on intestinal mucosal permeability was determined by detecting FITC-dextran fluorescence transmission in serum. FITC-labeled dextran was used as a permeability marker. The results show that the fluorescence intensity values in the FITC-70 group were significantly lower than those in the FITC-4 group, indicating that small molecules easily passed through the intestinal wall barrier, whereas large molecules did not easily pass through. After 4 h of drug gavage, The serum fluorescence intensity of the FITC-70+MO group was significantly stronger than that of the FITC-70 group (*p* < 0.05) (Figure 6A), while after 8 h of gavage, the serum fluorescence intensity of the FITC-70+MO group was significantly higher than that of the FITC-70 group (*p* < 0.01), which was consistent with the literature (Figure 6B). The serum fluorescence intensity of the FITC-70+MO group was significantly stronger than that of the FITC-70 group (*p* < 0.05) (Figure 6A), while after 8 h of gavage, the serum fluorescence intensity of the FITC-70+MO group was significantly higher than that of the FITC-70 group (*p* < 0.01), which was consistent with the literature (Figure 6B). It is tentatively hypothesized that *M. oleifera* protein can increase serum fluorescence intensity in mice and can increase intestinal permeability.

### 2.9. Effects of M. oleifera Leaves Protein on Tight Junction Protein Expression in the Small Intestinal Epithelium

**Figure 7:**
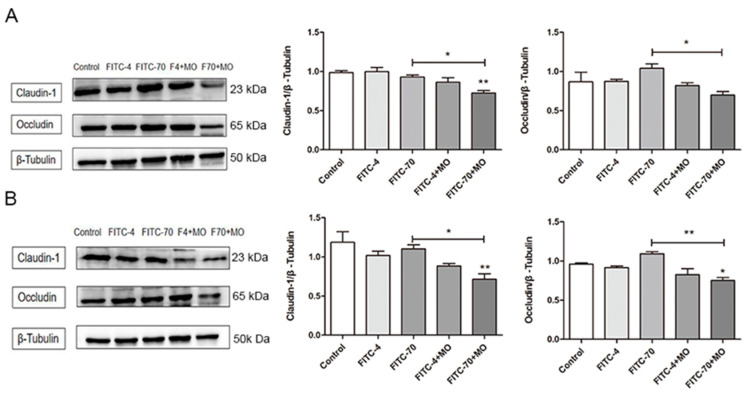
Expression of tight junction proteins in the small intestine. (**A**) Expression of tight junction proteins in the small intestine after *M. oleifera* leaves protein treatment for 4 h. Compared with the blank group, the FITC-70+MO group had a significant difference (** *p* < 0.01). Compared with the FITC-70 group, the expression of Occludin protein and claudin-1 protein was significantly decreased in FITC-70+MO group (* *p* < 0.05). (**B**) Expression of tight junction proteins in the small intestine after *M. oleifera* leaves protein treatment for 8 h. Compared with the blank group, the FITC-70+MO group had a significant difference (** *p* < 0.01).Compared with the FITC-70 group, the expression of Occludin protein (* *p* < 0.05) and claudin-1 protein(* *p* < 0.01) was significantly decreased in FITC-70+MO group. The data are presented as the mean ± SD (*n* = 10). Ordinary one-way ANOVA (Dunnett’s multiple comparisons test) was used. FITC-4: 10 mg/mL FITC-4; FITC-70: 10 mg/mL FITC-70; FITC-4+MO: 10 mg/mL FITC-4+MO (80 mg/mL); FITC-70+MO: 10 mg/mL FITC-70+MO (80 mg/mL).

The effects of *M. oleifera* leaves protein treatment on the expression of the tight junction proteins Occludin and Claudin-1 in the mouse small intestinal epithelium were further investigated with protein immunoblotting. The results in Figure 7A,B show that treatment with *M. oleifera* protein reduced the protein expression of Occludin and Claudin-1 in the mouse small intestinal epithelium compared to that in the blank control group. Specifically, the protein expression of Occludin and Claudin-1 in FITC-70+MO group was lower than that in FITC-70 group after 4 h (*p* < 0.01).

## 3. Discussion

Traditional theories suggest that changes in intestinal mucosal barrier permeability lead to antigen sensitization and mast cell-mediated allergic reactions, which are considered important in the development of food allergies. Mast cell degranulation increases intestinal permeability, but both T cells leading to mast cell degranulation and lectins leading to mast cell degranulation require passage through the intestinal epithelium. Through experiments using purely cultured intestinal epithelial cells, our experiment confirmed that *M. oleifera* leaves protein has the effect of altering inflammatory factor expression in the intestinal epithelium, an effect that likely leads to increased intestinal epithelial permeability. In addition, our in vivo mouse experiments showed that *M. oleifera* leaves protein could increase the fluorescence intensity of mouse serum at 4 h and 8 h and increased the permeability of FITC-labeled dextran. This effect does not depend on mast cell degranulation caused by T-cell differentiation or on the direct recognition of *M. oleifera* leaves protein by mast cells.

*Moringa oleifera* leaves protein has been extracted from fresh *Moringa oleifera* leaves by our research group before. It is a mixed protein, and the molecular weight of the protein is mainly 36 KDa and 55 KDa (Figure S1). Subsequent studies revealed that we get a 36 KDa and 55 KDa sequence (Figure S1). Extraordinarily, in a previous animal study on allergic reactions induced by *M. oleifera*, *M. oleifera* treatment caused increased intestinal vascular permeability in mice. Based on this evidence, we hypothesized that *M. oleifera* could increase intestinal permeability and conducted in vitro cell experiments to test this hypothesis. We used the Caco-2 cell line to establish a cell monolayer model and used this model to investigate whether *M. oleifera* leaves protein could impair Caco-2 cell tight junctions and thus increase intestinal permeability. It is well known that the formation and assembly of tight junctions are mediated by several proteins. Among them, three tight junction proteins, ZO-1, Occludin, and Claudin-1, are essential for maintaining intestinal cell tight junctions and limiting intestinal permeability [41,42]. It is generally believed that Occludin can act directly on ZO-1 that binds to the cytoskeleton, thus maintaining the stability of intestinal epithelial cell structure by regulating tight junctions via the cytoskeleton [43,44,45]. Claudins affect intestinal wall charges and increases or decreases in their expression alter the gaps between adjacent cells, affecting tight cell junctions and thus altering intestinal permeability [45]. 

*Moringa oleifera* leaves polysaccharides maintain the intestinal barrier to alleviate colitis [46], but here we have *Moringa oleifera* leaves protein, which has a different effect; some people who eat *Moringa oleifera* will experience allergies, and this may be caused by changes in intestinal permeability. This also increases the research significance of *Moringa oleifera* and our innovation. In this study, fluorescent labeling revealed that intestinal mucosal tissue in the *M. oleifera* leaves protein-treated group showed an overall disruption of continuity and disorganized structure, indicating abnormal distribution of each tight junction protein. In the Transwell monolayer assay, *M. oleifera* leaves protein decreased the electrical resistance and increased transmembrane fluorescence transmission in epithelial cell monolayers. Furthermore, the analysis of protein expression levels showed that different concentrations of *M. oleifera* leaves protein significantly decreased the expression of tight junction proteins to different degrees. Thus, overall, the results showed that the expression of tight junction proteins in Caco-2 cells was reduced, and that cell permeability was increased.

**Figure 8:**
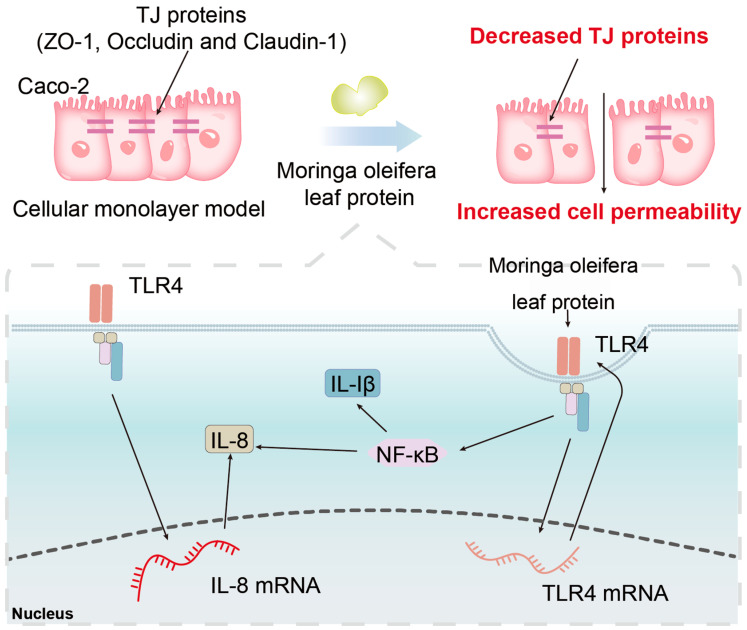
Schematic representation of the study on the effect of *M. oleifera* leaves protein on the barrier of Caco-2 cells. The effects of *M. oleifera* leaves protein on the expression of the tight junction proteins Occludin, Claudin-1, and ZO-1 in Caco-2 cells were further investigated. It was concluded that treatment with *M. oleifera* leaves protein significantly increased the mRNA expression of *IL-8* and *TLR4* in Caco-2 cells.

The present study shows that *M. oleifera* leaves protein increases intestinal permeability, but the mechanism used is not clear. A previous study has reported that the endopeptidase meprin can cleave tightly attached proteins and increase barrier permeability, thus disrupting the intestinal mucosal barrier [45]. Nonaka et al. [46] found that intracellular mannose-binding lectin may act as a transport carrier for proteins from the endoplasmic reticulum to the Golgi apparatus, controlling the formation of protein molecules. By screening the MAPK signaling pathway, Wang et al. [47] found that mannose-binding lectin can regulate cell proliferation and apoptosis by increasing its phosphorylation through the activation of P38. In the current study, it was concluded that treatment with *M. oleifera* leaves protein significantly increased the mRNA expression of *IL-8* and *TLR4* in Caco-2 cells. *TLR4* is a pattern recognition receptor that recognizes LPS and induces activation of *NF-κB,* which in turn promotes the expression of inflammatory cytokines such as *IL-1β* and *IL-8*, ultimately inducing an inflammatory response. All of the above pathways can differently regulate tight junction protein expression and affect barrier function, but how *M. oleifera* leaves protein functions remains unclear. Therefore, the specific mechanism by which *M. oleifera* leaves protein modulates tight junction protein expression needs to be further investigated. The next steps are to purify *M. oleifera* leaves protein allergens and explore how to eliminate *M. oleifera* leaves protein allergens and treat related allergies in order to provide a theoretical basis for the clinical prevention and treatment of protein allergic diseases. We aimed to show that the Moringa proteins have the effect of increasing intestinal permeability both in vivo and in vitro, and in vivo experiments confirmed this. The level of mRNAs were increased, but still not enough to prove that the receptor binds to the ligand, as *TLR4* may happen to be the target gene that activates the signaling pathway. This activated signaling pathway may or may not be *NFκB*, and the evidence is not sufficient at this point to make a definitive conclusion on this point (Figure 8). We have evidence that the *Moringa* leaves protein thereinto contains a unique phytoagglutinin that binds to numerous glycan-containing chain receptors on the surface of the cell membrane, and this is one of the reasons for the significant changes in mRNA. Our additional study demonstrates that the mode of action of LPS and *M. oleifera* leaves protein is different during dendritic cell maturation. We have always insisted that the changes that occur in purely cultured epithelial cells help us to find the truth, and that we can better observe the changes in the cells without the involvement of immune cells, which we hope are not affected by other factors. The in vivo study is simply to better verify that the same effect occurs for a short period of time as in the in vitro studies. Of course, for in vivo, if the epithelial cells secrete the relevant cytokines, the immune cells will be recruited and infiltrated to play a further role.

**Figure 9:**
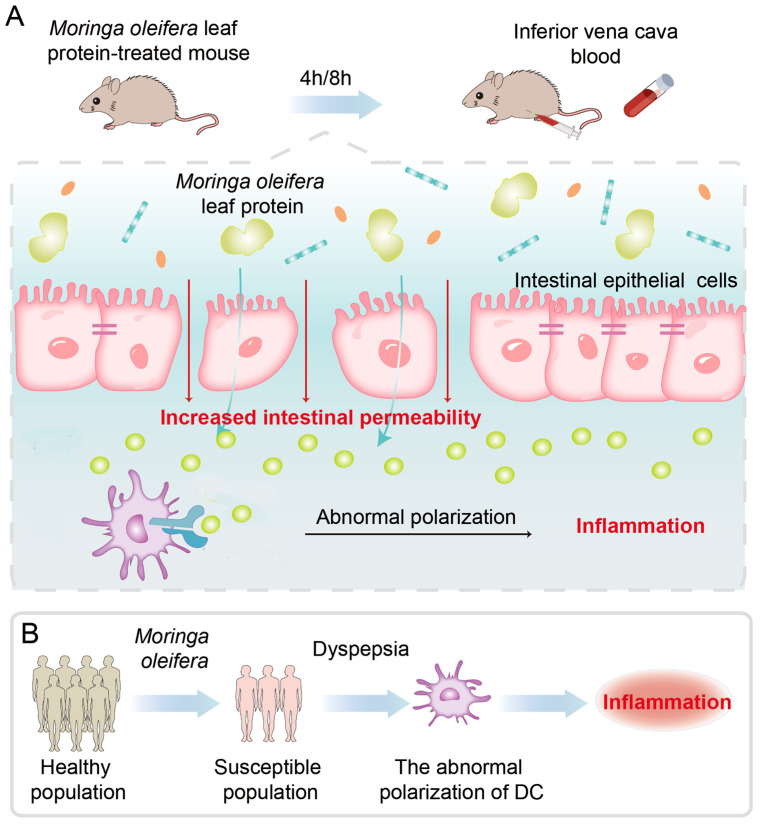
Schematic illustration of the study on the effects of *M. oleifera* leaves protein on the intestinal epithelial barrier and their mechanisms. (**A**) *M. oleifera* leaves protein increases the permeability of the intestinal barrier in mice, leading to abnormal polarization of DCs, which cause allergies to develop upon subsequent encounters with the allergens. (**B**) The increased permeability induced by *M. oleifera* leaves protein is a marker distinguishing susceptible and non-susceptible populations.

The effect of *M. oleifera* leaves protein on intestinal wall permeability was further tested in vivo in mice that were gavaged with FITC-70 kDa dextran + *M. oleifera* leaves protein (80–100 mg/mL) and then executed at 4 h and 8 h. Blood was collected from the inferior vena cava, and the fluorescence intensity in the serum was measured. Tight junctions consist of multiple protein complexes that form a barrier between adjacent cells and allow only specific small molecules to pass, such as water, electrolytes, and certain nutrients, while large molecules cannot pass [41]. FITC-dextran permeability is an important indicator of the barrier function of the intestinal mucosa. Therefore, we injected large-molecular-weight FITC-dextran into mice and evaluated intestinal permeability by measuring the amount of FITC-dextran that passed through the intestinal mucosal barrier into the blood circulation after 4 h and 8 h. Our results showed that *M. oleifera* leaves protein increased the fluorescence intensity of mouse serum at 4 h and 8 h, indicating that it increased the permeability of FITC-dextran. In addition, the expression of the intestinal mucosal tight junction proteins Occludin and Claudin-1 was decreased, and intestinal mucosal permeability was increased in mice, as detected using Western blot analysis. The primary roles of tight junctions are to seal cell gaps and regulate selective paracellular ion transport channels [48,49]. These junctions are composed of dynamic multiprotein assembly complexes and enable selective passage through intestinal epithelial barrier [50]. Damage caused by protein deletion or alteration leads to increased permeability between intestinal epithelial cells, allowing macromolecules such as bacteria, endotoxins, and food antigens to enter the circulation through the paracellular pathway and leading to the development of many diseases, including food allergies [51,52,53,54] (Figure 9A). There are many factors that can cause changes in intestinal permeability such as tacrolimus, zonulln, epidermal growth factor, microbial infections, and toxins that can cause tight junction changes [55,56,57]. The species richness of gut microorganisms, the amount of a certain microorganism, and the associated metabolites affect gut permeability; however, over a short period of time, such as 4 h or 8 h, related literature is rare, and there is not enough evidence to show whether our results indicate that changes in intestinal metabolites cause changes in intestinal permeability. As far as the available evidence is concerned, we prefer that which indicates that the active proteins of Moringa leaves cause changes in intestinal permeability.

The interactions between genetics and multiple environmental factors such as diet, drugs, and infections together control susceptibility to food allergies, which has been demonstrated to be a complex disease. Numerous studies on populations, families, and fraternal twins have shown that genetic factors are essential in disease development, although the specific molecular mechanisms of skin barrier dysfunction, and whether dysfunction of the systemic or local skin immune response to antigens or bacterial products plays a crucial role in the development of the disease, are not yet clear. Our findings reveal the existence of a susceptible population from the perspective of intestinal permeability. The increased intestinal permeability induced by *M. oleifera* leaves protein was a marker distinguishing susceptible and non-susceptible populations. These findings emphasize that it is necessary to study the pathogenesis of allergy at multiple levels, namely, genetics, skin barrier function, and the skin immune system, to provide a theoretical basis for the clinical diagnosis, prevention, and treatment of this disease (Figure 9B).

We conclude that *M. oleifera* leaves contain active proteins that increase intestinal permeability and reduce the expression of tight junction proteins. These proteins promote transmembrane transport in Caco-2 cells and increase intestinal permeability in test animals. It has been suggested that protein causes increased intestinal permeability via mast cell degranulation, and we investigated that *M. oleifera* leaves protein regulates intestinal permeability through immune cell-independent epithelial inflammation, leading to *M. oleifera* leaves protein allergy. Our findings regarding the increase in intestinal permeability caused by active *M. oleifera* leaves protein could aid in the prevention, diagnosis, and treatment of *M. oleifera* leaves allergy and provide new ideas for the development of oral vaccine adjuvants.

## 4. Materials and Methods

### 4.1. Materials and Chemicals

Fresh *M. oleifera* leaves were obtained from Tianyou Technology Co. of Dehong State. *M. oleifera* leaves protein was extracted according to reference [26]. Fresh moringa oleifera leaves were crushed and homogenized by adding phosphate buffer solution according to a ratio of material to liquid of 1:6, and placed in the refrigerator at 4 °C for 4 h, stirring once every 1 h. The residue was then filtered through gauze and the filtrate was centrifuged at 3500 r/min for 20 min using a centrifuge before the supernatant was collected. Then, 472 g of ammonium sulfate crystals were added to each 1 L sample solution for overnight precipitation, and the saturation of ammonium sulfate to completely dissolve the ammonium sulfate crystals was 70%. The samples were centrifuged again at 3500 r/min for 20 min, and the supernatant was discarded, leaving the precipitate. Then, PBS was added to dissolve the precipitate, and the precipitate was put into a dialysis bag for dialysis for 2 to 4 days until the dialysis water became clear. Finally, the dialyzed *M. oleifera* leaves’ protein was put into a freeze-drying dish for vacuum freeze-drying.

The Caco-2 human colon cancer epithelial cell line was purchased from the Cell Bank of the Chinese Academy of Sciences. Thiazole blue (MTT) was purchased from Sigma (St. Louis, MI, USA). Phosphate buffer solution (PBS), high-efficiency cell/tissue lysis solution (RIPA lysis buffer), dimethyl sulfoxide (DMSO), penicillin-double antibody, sheep anti-rabbit IgG-HRP, sheep anti-mouse IgG-HRP, and a two-color prestained marker were purchased from Beijing Solaibao Technology Co. (Beijing, China). BCA protein quantification kit was purchased from Beyotime (Beijing, China). An occludin antibody (ab216327) was obtained from Abcam (Cambridge, UK). A SYBR Green PCR kit and a reverse transcription kit (RR047A) were purchased from Takara Corporation (Beijing, China). Fluorescein isothiocyanate (FITC)-labeled dextran 4 kDa and 70 kDa were purchased from Shanghai Mao Kang Biotechnology Co. (Shanghai, China). All other reagents used in this work were of analytical grade.

### 4.2. Caco-2 Cell Assay

#### 4.2.1. Cell Viability Assay

Caco-2 cells were maintained and cultured in DMEM (Dulbecco’s Modified Eagle Medium: Biological Industries from Israel), and supplemented with 10% fetal bovine serum (Biological Industries, Beit-Haemek, Israel), streptomycin, and penicillin (100 mg/L, 100 U/L, respectively: Solaibao Technology Co. from Beijing, China). Cells were inoculated in 96-well culture plates at a density of 1.0 × 10^4^ cells/mL, 100 μL per well, and incubated in a 5% CO_2_ incubator at 37 °C for 24 h. After the cells had grown to 80% confluence, the original cell culture medium was discarded, and 25 μg/mL, 50 μg/mL, 100 μg/mL, 200 μg/mL, 400 μg/mL, 800 μg/mL, or 3200 μg/mL of *M. oleifera* leaves protein or the negative control (normal cell culture medium only) was added. Eight replicates were set for each concentration. After incubation for another 48 h, 100 μL of MTT reagent at 0.5 mg/mL was added to each well, and the cells were incubated for 4 h. An amount of DMSO equal to the amount of MTT (i.e., 100 μL) was added, and the absorbance value of each well was measured at a wavelength of 490 nm [25]. Cell viability was calculated as follows [26]:

Cell viability = A_490_ of the experimental group/A_490_ of the blank control group × 100%. (1)

#### 4.2.2. Establishment and Grouping of the Caco-2 Cell Permeability Model

Cells obtained using the above method were cultured and inoculated at a density of 5 × 10^5^ cells/well in the upper chamber of 24-well Transwell inserts (pore size 0.4 μm). After inoculation, the solution was changed every 2 days and changed daily after 1 week until 21 d, when the transmembrane resistance had stabilized [26,27].

#### 4.2.3. Evaluation of Cell Transepithelial Electrical Resistance (TEER)

The Caco-2 monolayers (5 × 10^5^ cells/500 mL) were cultured for 21 d on 24-well transwell inserts (Corning Inc. from New York, NY, USA; pore-size 0.4 mm; polycarbonate), and 25 μg/mL, 50 μg/mL, 100 μg/mL, 200 μg/mL, 400 μg/mL, 800 μg/mL, or 1600 μg/mL of *M. oleifera* leaves protein or the negative control (normal cell culture medium only) was added for different times, and the TEER values were measured. TEER was measured using Millicell ERS-2 Epithelial volt-ohm meter (EMD Millipore Corp. from MILLIPORE (Burlington, MA, USA)) and expressed in standard units as Ω.cm^2^.

TEER = (R − R_blank_) × A(Ω × cm^2^) (2)

where R is the actual measured resistance value, R_blank_ is the resistance value of the blank membrane without added cells, and A is the membrane area.

#### 4.2.4. Permeability Assays

Caco-2 monolayers, plated on the 24-well transwell inserts (Corning Inc. from New York, NY, USA), were allowed to grow for 48 h after the completion of the TEER measurements at its peak; cells were used to determine paracellular permeability. Briefly, 200 μL of tracer solution (4 kDa fluorescein isothiocyanate [FITC]dextran or 70 kDa- dextran-FITC, 1 mg/mL each; for detection of paracellular permeation) was added to the apical chamber of each insert for 2 h. The basal chamber was filled with 500 μL of DMEM medium. The inserts were incubated in the CO_2_ incubator to allow fluorescent tracer molecules to pass from the apical chamber to the basal chamber. Fluorescence was measured from basal chamber medium as an indicator of permeability, using a Varian Cary Eclipse Fluorescence Spectrophotometer.

#### 4.2.5. Determination of the mRNA Expression of Tight Junction Proteins by Real-Time PCR (RT–PCR)

To further investigate the effect of *M. oleifera* leaves protein on tight junctions in Caco-2 cells, we used fluorescence quantitative RT–PCR. Specifically, we detected the effects of different concentrations of *M. oleifera* leaves protein (100 μg/mL, 200 μg/mL, 400 μg/mL, 800 μg/mL, and 1600 μg/mL) on the mRNA expression of tight junction proteins in Caco-2 cells. Total RNA was extracted from Caco-2 cells using a Takara MiniBEST Universal RNA Extraction Kit (9767), and the RNA was reverse-transcribed into cDNA with a PrimeScript RT Reagent Kit with gDNA Eraser (Takara, RR047A). Amplification was performed using the synthesized first-strand cDNA as a template (according to the SYBR Green kit instructions), and gene expression was calculated as follows:

ΔCt = Ct _target gene_ − Ct_1RPL-19_, ΔΔCt = ΔCt _treatment group_ − ΔCt _control group_. (3)

The relative differences in target gene expression between samples were calculated with the 2^−ΔΔCt^ formula, and the primer sequences are shown in Table 1.

#### 4.2.6. Immunoblot Analysis

The effects of different concentrations (100 μg/mL, 200 μg/mL, 400 μg/mL, and 800 μg/mL) of *M. oleifera* leaves protein on the expression of the tight junction proteins Occludin, Claudin-1, and ZO-1 in Caco-2 cells were further investigated with protein immunoblotting. Proteins were extracted from Caco-2 cells using the prepared RIPA lysate, thawed on ice, mixed, and centrifuged transiently; RIPA lysates containing protease inhibitors were prepared, lysed of adherent cells, SDS–PAGE (the percentage of SDS was 10%), separated, and transferred to PVDF membranes. The membranes were blocked with a blocking solution (5% skim milk) at room temperature for 1 h. The membranes were incubated overnight at 4 °C with primary antibodies against ZO-1 (1:500), Claudin-1 (1:500), Occludin (1:600), and GAPDH (1:1000). The membranes were then washed with TBST, incubated with HRP-conjugated secondary antibodies (1:2000) at room temperature for 2 h, and washed again. The results were analyzed using an ECL Protein Blotting Detection System and ImageJ software (V1.8.0).

#### 4.2.7. Effect of *M. oleifera* Leaves Protein Treatment on Occludin Protein Localization in Caco-2 Cells

The Caco-2 monolayers (5 × 10^5^ cells/500 mL) were cultured for 21 d on 24-well transwell inserts. DMEM containing different concentrations of *M. oleifera* leaves protein was added for the required time. The cells were then rinsed 3 times with PBS (10 min each time), fixed with 4% paraformaldehyde for 15 min, rinsed with PBS, treated with 0.5% Triton X-100 for 10 min, rinsed with PBS, and blocked with 100 mL/L calf serum albumin for 1 h. A polyclonal rabbit anti-Occludin antibody was added at a dilution of 1:50, and the cells were incubated overnight at 4 °C. Next, the cells were rinsed with PBS, incubated with a FITC-labeled sheep anti-rabbit IgG antibody at 37 °C for 1 h, rinsed with PBS again, washed with distilled water, sealed with anti-fluorescence quenching blocker, and observed to assess the distribution of Occludin in the tight junctions of cells under a fluorescence microscope.

### 4.3. Animal Experiments

#### 4.3.1. Study Subjects and Subgroups

**Figure 10:**
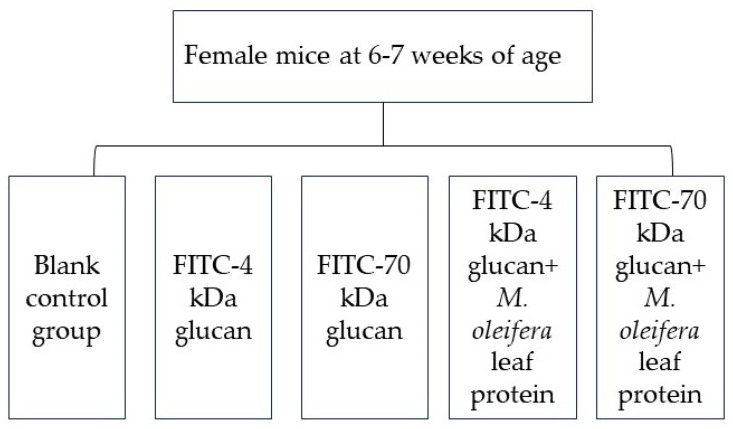
Groups for the animal experiments. Blank control group was given normal saline by gavage. The concentrations of FITC-4 kDa glucan and FITC-70 kDa glucan were both 10 mg/mL, for detection of paracellular permeation). *Moringa* leaves proteins were gavaged to mice at gavage dose of 0.3 g/100 g/B.W.

Female specific pathogen-free (SPF)-grade Kunming mice, aged 6–7 weeks (Kunming mice are smooth and the body shape is plump, and female mice from different batches are easy to be raised in groups), were purchased from Hunan Slaughter Jingda Laboratory Animal Co., Ltd. (Changsha, China), and housed at 25 ± 2 °C under a relative humidity of 40–60%. After one week of acclimatization, the mice were randomly grouped according to their body weight and gavaged as shown in Figure 10. And there were 10 mice in each group. All animal experiments were approved by the Life Science Ethics Review Committee (acceptance number: 202107021) and were performed strictly in accordance with the relevant laws and regulations, of the state and Yunnan Province, on the ethics and biosafety of experimental animal welfare.

#### 4.3.2. Mouse Serum Fluorescence Intensity Assay

According to the assay protocol of Hanash et al. [28], gavage was performed at 0.35 mL per mouse at doses according to the groups in Figure 10. After 4 h and 8 h, mice were anesthetized intraperitoneally with 10% chloral hydrate (0.3 g/100 g), and blood was collected from the eye of each mouse into an anticoagulation tube (1.5 mL centrifuge tube impregnated with sodium heparin) and centrifuged at 3000 rpm for 15 min to obtain the supernatant (i.e., plasma). Then, 200 μL was transferred to an enzyme labeling plate. The fluorescence intensity of each sample was measured in a multifunctional enzyme reader at an excitation wavelength of 480 nm and an emission wavelength of 520 nm.

#### 4.3.3. Assessment of Physiological Indicators in Mice

All experimental mice were sacrificed by the cervical dislocation method, and the abdominal skin was disinfected with 75% alcohol. The abdominal wall was cut along the abdominal midline, layer-by-layer with sterilized instruments, to expose the internal organs. The whole section of intestine from the anal opening to the ileocecal region was removed and spread on ice. The appearance of the small intestine was observed and recorded, the length of the small intestine was measured, and the contents of the small intestine were removed and washed with ice-cold saline. The liver, kidneys, spleen, stomach, thymus, and mesenteric lymph nodes of each mouse were weighed, and the organ index of each mouse was determined. Some of the intestinal tissue specimens were placed into lyophilization tubes, rapidly snap-frozen in liquid nitrogen, and then transferred to a −80 °C freezer for cryopreservation.

#### 4.3.4. Western Blot Analysis

After being heat-denatured in SDS-loading buffer and electrophoresed by 10% SDS–PAGE, the proteins in the samples were blotted to a PVDF transfer membrane (0.45 μm, Thermo Scientific™, Waltham, MA, USA), blocked with 5% skimmed milk dissolved in Tris-buffered saline for 1 h at room temperature, immunoblotted with the primary antibodies overnight at 4 °C, and incubated with HRP-conjugated secondary antibodies at room temperature for 1 h. Imaging was performed with an enhanced chemiluminescence detection kit and a Tanon-5200 Multi automatic image analyzer (Tanon, Shanghai, China)

### 4.4. Statistical Analysis

All the data in this study are presented as the means from three or more independent experiments ± the standard deviations (SEMs). The data were analyzed using GraphPad Prism software (7.0.0), and one-way analysis of variance (ANOVA) was used for comparisons among groups. * *p* < 0.05 indicates a statistically significant difference.

## Figures and Tables

**Table 1:** Primer sequences for gene expression analysis by RT–PCR.

Genes	Forward	Reverse
Occludin	CTTCCAATGGCAAAGTGAATG	TACCACCGCTGCTGTAACGAG
Claudin-1	CCAGGTACGAATTTGGTCAGG	TGGTGTTGGGTAAGAGGTTGT
ZO-1	GAGCCTAATCTGACCTATGAACC	TGAGGACTCGTATCTGTATGTGG
IL-8	CCTGAACCTTCCAAAGATGGC	TTCACCAGGCAAGTCTCCTCA
TLR4	GTACCTGGGGAACAACCTCTT	GCAGCTTGACTAGACTCTCCA
1RPL-19	GAAGGTCAAAGGGAATGTGTTCA	CCTTGTCTGCCTTCAGCTTGT

## Data Availability

The datasets presented in this study can be found in online repositories. The names of the repository/repositories and accession number(s) can be found in the article.

## Supplementary Materials

The supporting information can be downloaded at: https://www.mdpi.com/article/10.3390/ijms242216425/s1.
