# Supplementary material for: Moringa oleifera Leaves Protein Enhances Intestinal Permeability by Activating TLR4 Upstream Signaling and Disrupting Tight Junctions
Source: Int J Mol Sci. 2023 Nov 16;24(22):16425. doi: 10.3390/ijms242216425 (PMC10671199; doi:10.3390/ijms242216425)
Supplement: Supplementary file 1 [file ijms-24-16425-s001.zip › ijms-2633731-supplementary.pdf]

Figure Supplementary S1

Moringa oleifera leaf by our research group before. It is a mixed protein, and the molecular weight of the protein is mainly 36 KDa and 55 KDa, Subsequent studies revealed that degradation of 36 kDa to 23 kDa was found as shown in the Figure Supplementary S1.

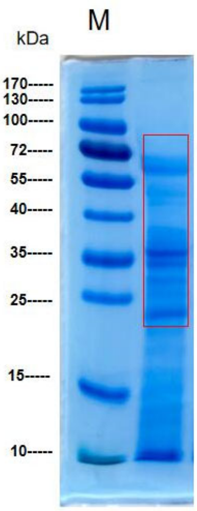

Figure Supplementary S1. SDS–PAGE of *Moringa oleifera* leaf protein

And we get the 55 KDa following sequence:
RIDEIRRERASLVPGANGNGGTIVDSGSTFTFIFDMKNVGVATPTLQEWSDEAGPSSSLQNHLHPT
DPNTKIGGLDSFV
RVFDVDTNATIATLEAPPSEVWGMQFEGRTNPSSSSQHLIHLEHEFSAHNYHPVPIVFSQAKGSIIC
AMSHYGFEIVQT
LIVDIEPDVHDPEGNKYLDFDPKGTTLAVAGGGSASVKLWDTATWKLIATLSIPRPEGPKPTDKS
GSKKFVLSVAWSP
DGKRVAMEKLEVQHSHVEVRGLKLHVAEVGTGPKVVLFLHGFCGSMDGTICVFDVDRAKFLH
HLEGHYMPVRSLV
YSLLEQVEALGESGKVDEAEALPVDPRVLFSASDDTHVQMYDAEGKSLIGAMSGHSSWVLSVDA
SPDGAAIATGSS
DRTVRLWDLKMRAAVQTMSNHTDQVWSVAFRPPGGTGVRAGRLASVSDDKSISLYDYSRCGRN
LYDSSDKDI

And we get the 36 KDa following sequence:
DNVLFEGILLKPSMVTPGAESKDRATPEKVAEYTLKLLHQRIPPAVPGIMFLSGGQSEVEATLNLN
AMNQGPNPWHVSFSYARALQNTCLKTWGGRPENVKAAQDALLIRAKANSLAQLGKYTGEGES
EEAKKGMFVKGYTY
